# Detection of *Salmonella* and *Escherichia coli* along the Fish Value Chain in Bahir Dar City, Ethiopia

**DOI:** 10.1002/puh2.204

**Published:** 2024-06-30

**Authors:** Temesgen Sendekie Ayalew, Habtamu Tassew Tarekegn, Belayneh Getachew Ayalew

**Affiliations:** ^1^ School of Animal Science and Veterinary Medicine Bahir Dar University Bahir Dar Ethiopia; ^2^ National Veterinary Institute Bishoftu Ethiopia

**Keywords:** Bahir Dar, fish, lake, retailers, swab, virulence

## Abstract

**Background:**

Fish is a possible source of foodborne infections with *Salmonella* and *Escherichia coli*. This study was conducted to identify *Salmonella* and *E. coli* along the fish value chain in Bahir Dar City, Ethiopia.

**Methods:**

A cross‐sectional study was undertaken with purposive sampling. A total of 121 specimens comprising fresh fish, retailing fish, filleted and cooked fish, swabs, and water samples were collected. Both culture based and molecular methods were used for detection.

**Results:**

*E. coli* isolated from 41 (33.88%) and *Salmonella* from 6 (4.96%) specimen. The highest *E. coli* isolation rate was from retailing fish 16 (80%), whereas the highest *Salmonella* isolation rate from filleted tissue 2 (20%). At restaurants, 12 (30%) samples were positive for *E. coli* and 3 (7.50%) for *Salmonella*. All 41 *E. coli* isolates were resistant to amoxicillin/clavulanate, whereas no resistance was shown for gentamicin and amikacin. Two thirds of *Salmonella* isolate and 95.12% of *E. coli* were detected as they develop multidrug resistance. The highest rate of resistance was recorded for ceftazidime against all (*n* = 6) isolates of *Salmonella* species. From a total of 10 *E. coli* isolates tested by PCR, 4 were positive for hemolysin A1 and/or *eae* virulence genes.

**Conclusions:**

The study detects the potential biological hazards along the value chain. Hygiene of fish handlers and their working environment and proper fish cooking are highly valuable. One health campaign should be carried out on drug resistance, contamination of the lake, and fish safety.

## Graphical abstract

A cross‐sectional study was undertaken to identify *Salmonella* and *Escherichia coli* along the stages of the fish value chain. There was a serious hygienic issue from the producer to the consumer level, with a special concern at the site of retailer.

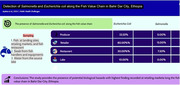

## 1 Background

Pathogens are among the primary biological dangers to consumers of aquatic foods [1]. *Salmonella* and *Escherichia coli* species together with *Staphylococcus, Clostridium*, *Campylobacter*, *Listeria*, *Vibrio*, and *Bacillus* are causative agents for more than 90% of food poisoning diseases [2, 3]. Foodborne infections can be found throughout the supply chain, and risk‐based approaches place a lot of emphases on figuring out where these pathogens come from. The surfaces of handling equipment, as well as storage and washing water inside the production environment come into contact with the fish throughout the value chain, from capturing to landing and processing. Microorganism contamination during this interaction could happen due to the water, the staff, or insufficient cleaning practices [4].

Customers of restaurants and street food are at danger of contracting enteropathogens through poor hand hygiene and food handling, which may result in fecal contamination of foods by hands during meal preparation, especially in low‐income nations [5, 6, 7]. A fish's hygienic status is determined by the presence of *Enterobacteriaceae* such as *E. coli*, which serve as markers of possible pathogen presence [8].

In Ethiopia, only few studies have been conducted to reveal the occurrence of *E. coli* and *Salmonella* spp. in fish. The bacteria *E. coli*, *E. coli* O157:H7 (predominant and most virulent serotype of *E. coli*), and *Salmonella* spp. from fish were reported [9, 10, 11, 12, 13]. Fish processing takes place around Lake Tana mostly in a traditional system, on bare ground in unsanitary conditions for gutting and filleting with little to no consideration for safety. The fish are gutted and cleaned before being carried to local markets, restaurants, and hotels in Bahir Dar City. Fish processing is not meeting the criteria required for hazard analysis critical control point system compliance [14].

In the study conducted at the upper Blue Nile River watershed in Northwest Ethiopia, *E. coli and Salmonella* are among the contaminants of fish in the study area, and their occurrence in fish could represent a risk to consumers [11]. Further studies such as molecular works on *E. coli* are recommended to show the public health importance of the pathogen [10]. Furthermore, the antimicrobial resistance (AMR) pattern of those possible pathogens in the fish value chain, as well as the source and level of contamination, needs also be addressed. Therefore, the study was conducted to identify *Salmonella* and *E. coli* with its common virulence genes and their AMR pattern along the stages of the fish value chain in Bahir Dar city, Ethiopia.

## 2 Methods

### 2.1 Study Design and Setting

**FIGURE 1:**
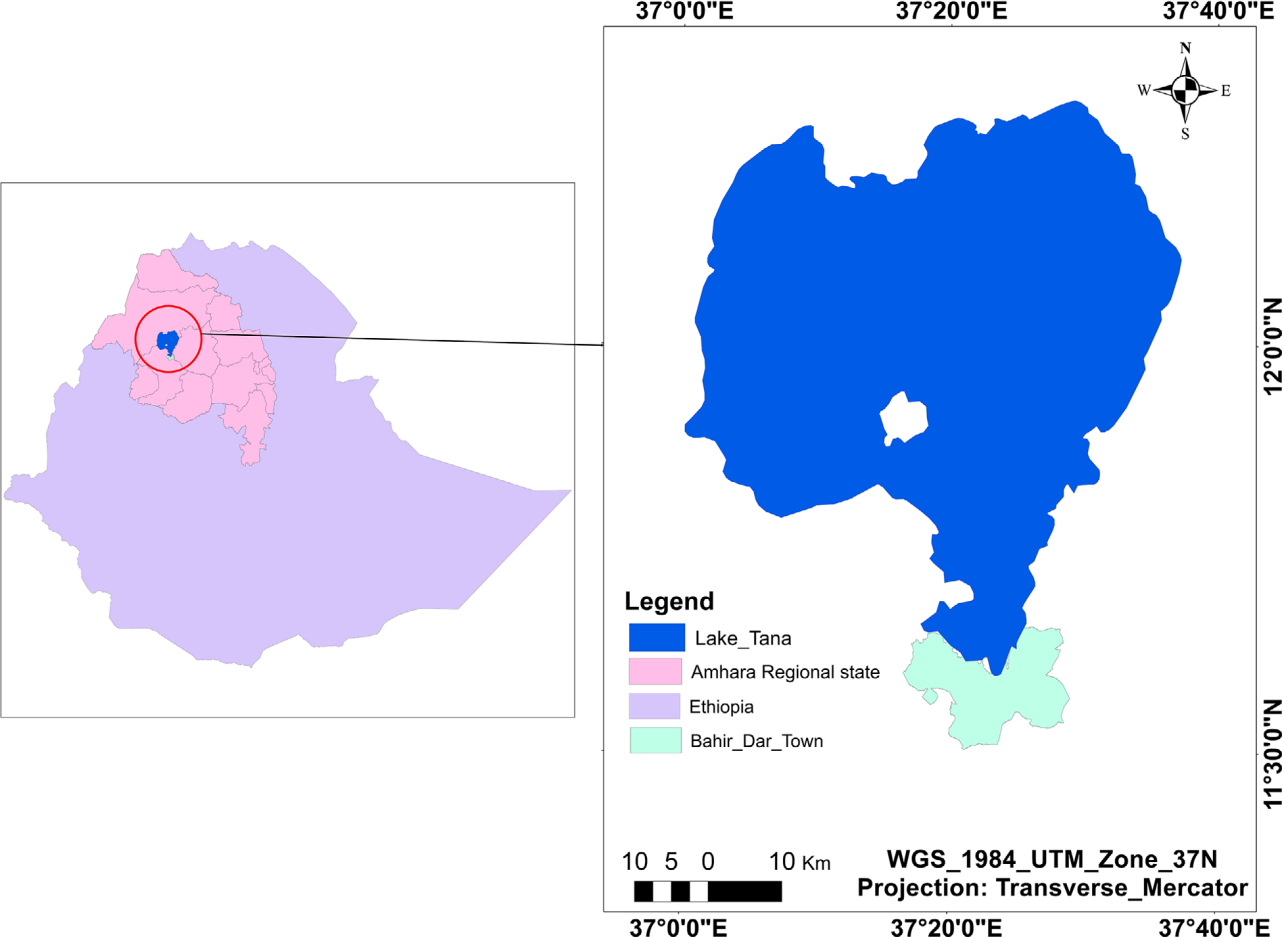
Arc GIS product for map of the study area (Bahir Dar City and Lake Tana, 2023).

A cross‐sectional study was undertaken at Bahir Dar landing sites of Lake Tana (Figure 1) Bahir Dar, Ethiopia. Administratively, Bahir Dar is a Special Zone and one of the leading tourist destinations in Ethiopia, with a variety of attractions in the nearby Lake Tana and Blue Nile River. The city is divided into 9 subcities and 32 rural *kebeles*, comprising an area of 213.55 km^2^. Data taken from Ethiopian Central Statistics Agency indicated that the study area had a total population of 348,429 in 2017 [15].

Lake Tana is the largest lake in Ethiopia. It embraces 50% of the country's fresh water. The resources are exclusively significant for the surroundings, industry, domestic, and agricultural purposes [16].

The fish of Lake Tana show impressive diversity and unprecedented uniqueness. Fish production from capture fisheries includes Nile Tilapia (*Oreochromis niloticus*), Large African Barbs (*Labeobarbus* spp.), and African Catfish (*Clarias gariepinus*) from the lake [17].

### 2.2 Sampling Strategy

Two landing sites (Bata and Shimbit Mikael) and 10 restaurants were selected with purposive sampling based on their better capacity to supply the harvested fish for the city and their higher number of consumers to serve, respectively. Each visit to the landing sites resulted in the sampling of 5–6 harvested fish as a pooled sample from randomly selected containers carried on the boat. At the restaurant, swabs of both personnel's hands and equipment, including knives and cutting boards to be involved in fish preparation, were sampled. The swab of knives and cutting boards were mixed into a single‐transport media as a single sample. Water samples were collected from five fish harvesting sites of the lake at morning time.

### 2.3 Specimen Collection

Between February and November 2022, 10 visits were carried out for the collection of proposed specimens along the fish value chain (landing sites, retail markets, and restaurants in addition to water samples from the lake) randomly.

**FIGURE 2:**
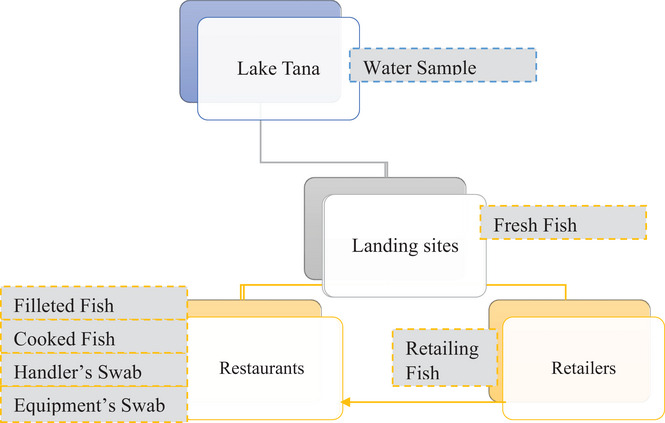
The schematic illustration of sampling channels with respective specimen types along the fish value chain in Bahir Dar City, Ethiopia, 2023.

To accommodate the existing fish value chain in Bahir Dar City, seven types of specimens were included (Figure 2).

A total of 121 specimens comprising 51 fresh fish, 20 retailing fish, 20 filleted and cooked fish (each 10), 20 swabs from processing equipment and hand of food handlers (each 10) and 10 specimens of water were collected from 2 fish landing sites in the southern gulf of Lake Tana, retail fish markets, and 10 fish restaurants in the city as illustrated in Figure 2.

### 2.4 Bacterial Enumeration

Bacterial Enumeration was conducted by using separate sterile pipettes, decimal dilutions of 10^−1^ to 10^−8^ of food homogenate and standard methods were followed to grow the countable colony for each of the counting type. Total plate count agar, Violet Red Bile agar, and eosin methylene blue (EMB) agar were used to estimate total viable count (TVC), total coliform count (TCC), and *E. coli* count, respectively. Finally, the number of colonies were counted with a colony counter for each plate having colonies between 30 and 300 and multiplied by the dilution factor to calculate the total colony forming units per gram of (CFU/g) the sample [18].

### 2.5 Isolation of *Salmonella* species and Isolation of *Escherichia coli*

Aseptically 25 g sample was mixed with 225 mL of sterile Buffered Peptone Water (BPW) in a sterile plastic bag and blended with a stomacher for 2 min. The BPW homogenate then was incubated for 24 h at 37°C and processed with standard procedure of isolation of *Salmonella* and *E. coli*.

The suspected colonies for *Salmonella* spp. with pink with a black center on xylose lysine deoxycholate and for *E. coli* with green metallic sheen on EMB agar were cultured on nutrient agar and biochemically confirmed by the urease test, triple sugar iron agar slants, and Indole, Methyl Red‐Voges Proskauer, and Simmons Citrate agar tests (IMViC tests). The result was then recorded as presumptive *Salmonella* and *E. coli* based on their standard biochemical characteristics. The presumptive isolates were then stored at −20°C for further molecular detection (PCR).

The antibiotic sensitivity test was done by using the standard Kirby–Bauer disk diffusion method [19]. Ten chemotherapeutic agents (antibiotic discs) for *E. coli* isolates and six discs for *Salmonella* isolates were selected and used as described by the CLSI 2020 (Clinical and Laboratory Standards Institute) guideline with their availability [20]. The zone size of inhibition for each drug was indicated on the recording sheet as susceptible (S), intermediate (I), or resistant (R) based on the interpretation chart [20].

Genomic DNA extraction and purification were performed as per the protocol given by Genomic DNA Purification Kit (Qiagen) available at National Veterinary Institute (NVI) for *E. coli* isolates. The purity of extracted DNA was examined with 2% agarose gel. The selected 10 *E. coli* isolates were subjected to PCR for the detection of 3 pathogenic genes (EVT1, *eae*, and hemolysin A [hlyA]). The standard methods were followed to conduct PCR.

Amplified PCR products were analyzed by gel electrophoresis at 120 V for 80 min in 2% agarose containing 4 μL Gel red (Biotium) with standard methods. The products were visualized with UV illumination and imaged with a gel documentation system (UVtec 08 100554). The expected size of the PCR products flanked by the primer pairs were around 350 bp for *eae*, 1551 bp for *hlyA* gene, 349 bp for *VT1*.

### 2.6 Data Management and Analysis

The STATA/SE (special edition) 17 and Microsoft Office Excel 2007 were used as statistical tools for data processing and analysis. Data collected were entered in an Excel spread sheet. Descriptive statistical analysis was used for variables under study, particularly with frequency distributions. Bacterial count data were transformed into logarithm values (log10 CFU/mL) before statistical analysis. To establish statistically significant differences between means of microbial loads in stages of the value chain, the one‐way analysis of variance was applied by using STATA/SE17.

### 2.7 Ethical Considerations

This study was carried out in accordance with the Declaration of Helsinki and approved by the Institutional Review Board (IRB) or Ethics Committee of Bahir Dar University, Ethiopia. The entire sample collection was done after informed verbal consent by each participant about the objectives of the study, benefits, and their rights.

## 3 Results

### 3.1 Bacterial Enumeration

The mean TVC, TCC, and *E. coli* count of the samples were 8.44, 5.99, and 4.05 log_10_ CFU/g, respectively. The details are given in Table 1.

**TABLE 1:** Bacterial counts from different samples along the fish value chain in Bahir Dar City, Ethiopia, 2023.

Sample type	Mean TVC (log_10_ CFU/g or mL)	Mean TCC (log_10_ CFU/g or mL)	Mean *Escherichia coli* count (log_10_ CFU/g or mL)
Fresh fish	8.58	6.19	4.45
Retailing fish	8.88	6.11	4.02
Filleted fish	8.90	5.45	2.93
Cooked fish	6.21	4.60	3.07
Lake water	9.95	9.21	6. 65
**Total**	**8.44**	**5.99**	**4.05**

Abbreviations: TCC, total coliform count; TVC, total viable count.

**FIGURE 3:**
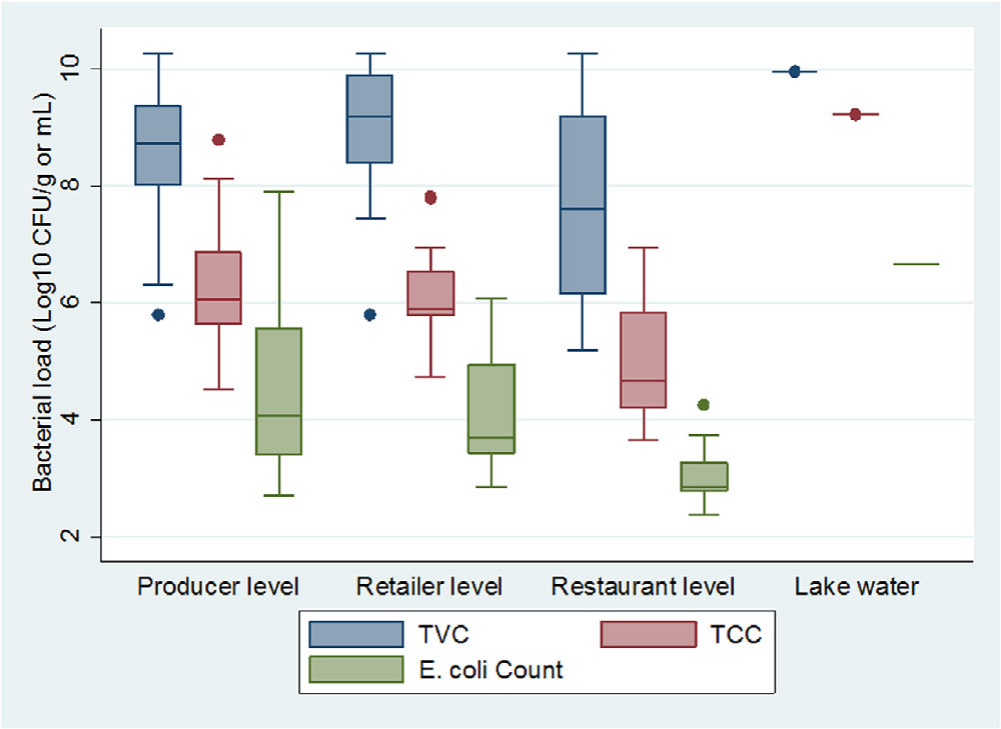
A box plot distribution of bacterial count data set along the fish value chain in Bahir Dar City, Ethiopia, 2023.

The result of bacterial enumeration showed that an ascending order from TVC to TCC and *E. coli* count as shown in Figure 3.

The highest mean was recorded from the lake for all TVC, TCC, and *E. coli* count. The second highest mean count resulted at the retailer level for TVC and restaurant level for both TCC and *E. coli* count. The details are given in Table 2.

**TABLE 2:** One‐way analysis of variance (ANOVA) output of stages of food chain on total viable count (TVC), total coliform count (TCC), and *Escherichia coli* count along the fish value chain in Bahir Dar City, Ethiopia, 2023.

Types of count	Source of variation	Sum of squares	Degree of freedom (df)	Mean square (S^2^)	F‐Ratio	Prob > *F*
TVC	Between	24.6419277	3	8.21397589	6.65	**0.00**
	Within	106.278034	86	1.2357911		
	Total	130.919962	89	1.47101081		
TCC	Between	41.5537649	3	13.851255	16.18	**0.00**
	Within	76.1991702	89	0.856170451		
	Total	117.752935	92	1.27992321		
*E. coli* Count	Between	31.1981652	3	10.3993884	7.36	**0.00**
	Within	70.6732634	50	1.41346527		
	Total	101.871429	53	1.92210243		

Bold values indicate the best statistically significant findings.

*E. coli* was isolated from 41 (33.88%) specimens and *Salmonella* from 6 (4.96%) of specimen.

The result in Table 3 has shown the detailed findings across different sample types, stages of the fish value chain, and species of fish.

**TABLE 3:** The frequency of *Escherichia coli* and *Salmonella* isolates across specimen types along the fish value chain in Bahir Dar City, Ethiopia, 2023.

		*E. coli*		*Salmonella*	
Variables	Total examined	Positive	%	Positive	%
**Specimen type**					
Fresh fish	51	12	23.53	0	0.00
Retailing fish	20	16	80.00	3	15.00
Filleted fish	10	6	60.00	2	20.00
Cooked fish	10	3	30.00	0	0.00
Equipment swab	10	2	20.00	0	0.00
Handler's swab	10	1	10.00	1	10.00
Water	10	1	10.00	0	0.00
**Total**	**121**	**41**	**33.88**	**6**	**4.96**
**Stages of the fish value chain**					
Producer	51	12	23.53	0	0.00
Retailer	20	16	80.00	3	15.00
Restaurant	40	12	30.00	3	7.50
Lake	10	1	10.00	0	0.00
**Total**	**121**	**41**	**33.88**	**6**	**4.96**
**Species**					
*Oreochromis niloticus*	43	15	34.88	1	2.32
*Labeobarbus* spp.	16	8	50.00	2	12.50
*Clarias gariepinus*	10	4	40.00	0	0.00
Unidentified	52	14	26.92	3	5.77
**Total**	**121**	**41**	**33.88**	**6**	**4.96**

### 3.2 Antimicrobial Sensitivity Test

#### 3.2.1 *Escherichia coli* Isolates

The overall antimicrobial sensitivity test findings for presumptive *E. coli* isolates were given in Table 4.

**TABLE 4:** Antimicrobial sensitivity pattern of *Escherichia coli* isolates along the fish value chain in Bahir Dar City, Ethiopia, 2023.

Antimicrobial disc	Resistance isolates	%	Intermediate isolates	%	Susceptible isolates	%
Ciprofloxacin	5	12.20	6	14.63	30	73.17
Azithromycin	13	31.71	0	0.00	28	68.29
TMP‐Sulfa	5	12.20	0	0.00	36	87.80
Tetracycline	36	87.80	0	0.00	5	12.20
Amox/Clav	41	100.00	0	0.00	0	0.00
Ceftazidime	39	95.12	2	4.88	0	0.00
Ceftriaxone	22	53.66	13	31.71	6	14.63
Gentamicin	0	0.00	0	0.00	41	100.00
Amikacin	0	0.00	0	0.00	41	100.00
Nalidixic acid	1	2.44	28	68.29	12	29.27

The antimicrobial sensitivity test was analyzed for multidrug resistance rate (MDR). All 41 isolates showed a resistance against at least two discs and with a maximum MDR against six drugs by three of the isolates (Table 4). The MDR rate for the *E. coli* isolates was 95.12%.

### 3.3 *Salmonella* Isolates

**FIGURE 4:**
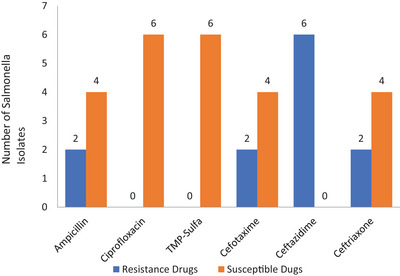
The antimicrobial sensitivity test result of *Salmonella* isolates along the fish value chain in Bahir Dar City, Ethiopia, 2023.

The highest rate of resistance was recorded for ceftazidime against all (*n* = 6) isolates of *Salmonella* species, whereas ciprofloxacin and TMP‐Sulfa were identified as having no resistance (Figure 4). One third of *Salmonella* isolates were detected as they develop MDR.

### 3.4 Virulence Detection

#### 3.4.1 *Escherichia Coli* Isolates

From the total 10 isolates tested by PCR, 4 were positive for 2 virulence genes (hlyA1 and eae) of *E. coli*. Among the three virulence genes of interest of *E. coli* for this study, only the two are detected (Table 5) whereas no VT1 gene was identified. A single *E. coli* isolate was detected with the two virulence genes.

**TABLE 5:** The description of positive samples for detected virulence genes along the fish value chain in Bahir Dar City, Ethiopia, 2023.

Sample Id_	Gene detected	Sample type	Stages of fish value chain	Species of fish
50	hly A1	Retailing fish	Retailer	Nile Tilapia
55	hly A1	Cooked Fish	Restaurant	Unidentified
68	eae	Filleted fish	Restaurant	Unidentified
72	hly A1and eae	Filleted fish	Restaurant	Unidentified

**FIGURE 5:**
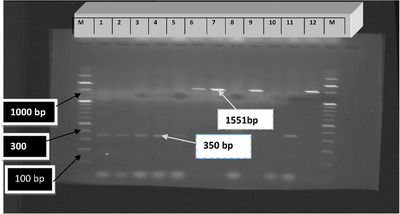
Agarose gel electrophoresis of virulence genes of the potential pathogenic *Escherichia coli* isolates along the fish value chain in Bahir Dar City, Ethiopia, 2023. The first and the last lane denoted by “M” (molecular ladder started at 100 bp), Lanes 1–9: for tested samples, Lane 10: negative control, Lane 11: positive control of eae gene—350 bp, Lane 12: positive control of Hly A1 gene—1551 bp.

The molecular work for 10 *E. coli* isolates revealed the presence of intimin gene (eae) in the 2 isolates and hlyA in the 3 samples as shown in Figure 5.

## 4 Discussion

The current study revealed that the mean TVC of the processed samples was 8.44 log_10_ CFU/g. This was much higher than the study undertaken in Turkey with a count range of 2.68–2.92 and 2.79–2.87 log_10_ CFU/g analyzed in the morning and evening, respectively [21]. The study conducted in Sri Lanka [22] with TVC of 6.30 log_10_ CFU/g and in Nigeria [23] ranged from 6.30 to 6.97 log_10_ CFU/g, which were lower than the present study.

The result of TCC was 5.99 log_10_ CFU/g is also higher than those of studies carried out in Turkey [21] with an average coliform count was 2.25–2.34 log_10_ CFU/g. The higher count might be a result of unhygienic handling and processing methods, such as utilizing filthy tools and containers with unwashed hands, undercooking, and/or postcooking contamination at the level of consumers.

The TVC mean count was higher but the load pattern along the sites of the value chain was in‐line with the result studied in Unguja Island at producer 5.85 log_10_ CFU/g, retailer 5.96 log_10_ CFU/g and consumer/restaurants 4.79 log_10_ CFU/g and Pemba Island Producer level 5.74 log_10_ CFU/g, retailer level 5.86 log_10_ CFU/g, and restaurant level l4.79 log_10_ CFU/g in Tanzania [24].

The bacterial count for cooked ready‐to‐eat fish in restaurants possessed the mean TVC (6.21 log_10_ CFU/g), which was higher than the study conducted in the upper Blue Nile watershed, Ethiopia reported as 4.90 log_10_ CFU/g [11] and the *E. coli* count (3.073 log_10_ CFU/g) was lower than the findings of the study in Northwest, Ethiopia, which is reported as 4.44 log_10_ CFU/g [10]. According to the standard set by Microbiological Guidelines for Food Safety, the mean TVC finding for cooked fish showed that it was at the borderline (between 4 and 7 log_10_ CFU/g) and the mean *E. coli* count was unsatisfactory (>2 log_10_ CFU/g) for consumers [25]. The mean TCC of cooked fish (2.07 log_10_ CFU/g) was much higher than the mean TCC found by the study done in the upper Blue Nile watershed (2.07 log_10_ CFU/g) [11]. Persons who are doing as retailers and processors with less awareness about hygiene and sanitation are important for the contamination of the fish [26].

Out of the total of 121 samples examined by biochemical tests in this study, *E. coli* was isolated from 41 (33.88%). This finding was higher than the study reported in the southern gulf of Lake Tana (2.4%) [9], Northwest Ethiopia (20%) [10], Hawassa (21%) [27] (23.3%) [12], Lake Zeway (12%) [28], Ghana (6.1%) [29], and Tanzania (8.6%) [30]. The result was however almost in‐line with the study undertaken in Northeast Algeria (32%) [31] but lower than the report in India (55.56%) [32], Egypt (42.2%) [33], and Sri Lanka (68%) [22].

The isolation rate of *Salmonella* 6 (4.96%) in this study was lower than the findings in different parts of Ethiopia (36.4%) [34], (58%) [27], (14.5%) [35], Nigeria (11.5%) [36], India (49.99%) [32], but it was almost in agreement with the reports in Ethiopia (6.82%) [11] and Brazil (4.0%) [37] and (3.4%) [38]. The variation might be attributed to the difference in identification technique, sample type, fish species, and most importantly the hygienic status of fishing, marketing, and processing environment.

Among the 10 analyzed handler's swabs at fish restaurants, 1 (10%) was positive for *Salmonella*. This might be because they do not wash their hands after having close contact with some animals. The result showed that the hygienic handling of fish could be taken as hazardous for consumers.

Out of the total 10 water samples taken from the lake 10% (*n* = 1) *E. coli* isolation rate has resulted. The report of the current study from the lake water was less than the study conducted in Cote d'Ivoire 51 (45%) [39]. This might be because of the number of water samples, the hygiene of the water bodies, and the identification technique. Pathogenic bacteria or alterations in the environment's natural micro flora could be a key sign of potential contamination [40]. The presence of *E. coli* in water shows fecal contamination [41].

The virulence detection study found that 4 *E. coli* isolates harbored virulence gene eae (*n* = 1) at the restaurant, hlyA at retailer (*n* = 1) and restaurant (*n* = 1), and both eae and hly A (*n* = 1) at restaurant. The study conducted by Al Qabili and his colleagues from edible fish in Egypt detected eaeA gene which was in agreement with the current study, but the additional stx genes were also identified which is not detected in the present study [33], whereas no pathogenic virulence gene was detected at the study in fish in Cote d'Ivoire [39] and Morocco [42].The eae gene's existence indicates a potential contributing factor to diseases that attach and expel the intestinal epithelial lining [43]. After confirming the coexistence of the stx gene, which is only present in Shiga toxin‐producing *E.coli* (STEC), it is critical to identify the eae gene from an *E. coli* isolate to discriminate between Enteropathogenic *E.coli* (EPEC) B and STEC pathotypes [44, 45, 46, 47].

Enterohemolysin (hlyA) gene might be directly related to how pathogenic the STEC strain is and how likely it is to infect individuals with more severe diseases [48]. Enterohemolysin causes epithelial and endothelial cell injury [49], and it can lyse erythrocytes to release iron [50]. As a recommendation a regular fish safety monitoring framework should be designed and implemented. Interdisciplinary (One Health) approach should be carried out against drug resistance, contamination of the lake, and fish safety issues.

### 4.1 Limitations

The molecular investigation was taken place for only 10 of the *E. coli* isolates that would be as a result of shortage of reagents. The sampling strategy used was purposive sampling that is a type of nonprobability sampling. The perception of fish handlers and concerned individuals was not addressed.

## 5 Conclusion

The bacterial load counts and identification of the pathogens along the fish value chain revealed that there is a serious hygienic issue with a special concern at the site of retailer. The possible sources of contamination of fish meat in the city were the lake water itself, the equipment used for fish handling and processing, and/or the fish handlers themselves. The findings of this study indicated that there are alarming biological hazards for fish handlers, consumers, researchers, and health organizations concerned with safeguarding public health.

## Author Contributions

Temesgen Sendekie Ayalew conceived and designed the analysis, collected the data, performed the analysis, wrote, and revised the paper. Habtamu Tassew Tarekegn and Belayneh Getachew Ayalew also conceived and designed the analysis, contributed to analysis tools, wrote, and revised the paper.

## Conflicts of Interest

The authors declare that there are no conflicts of interest regarding the publication of this paper.

## Data Availability

The data that support the findings of this study are available on request from the corresponding author.
